# Automatic Seizure Detection Based on Nonlinear Dynamical Analysis of EEG Signals and Mutual Information

**DOI:** 10.32598/bcn.9.4.227

**Published:** 2018-07-01

**Authors:** Behnaz Akbarian, Abbas Erfanian

**Affiliations:** 1. Iran Neural Technology Research Centre, Iran University of Science and Technology, Tehran, Iran.; 2. Department of Bioelectrical Engineering, School of Electrical Engineering, Iran University of Science and Technology, Tehran, Iran.

**Keywords:** Epilepsy, Mutual information, Nonlinear analysis, Recurrence quantification analysis, Seizure detection

## Abstract

**Introduction:**

In this paper, nonlinear dynamical analysis based on Recurrence Quantification Analysis (RQA) is employed to characterize the nonlinear EEG dynamics. RQA can provide useful quantitative information on the regular, chaotic, or stochastic property of the underlying dynamics.

**Methods:**

We use the RQA-based measures as the quantitative features of the nonlinear EEG dynamics. Mutual Information (MI) was used to find the most relevant feature subset out of RQA-based features. The selected features were fed into an artificial neural network for grouping of EEG recordings to detect ictal, interictal, and healthy states. The performance of the proposed procedure was evaluated using a database for different classification cases.

**Results:**

The combination of five selected features based on MI achieved 100% accuracy, which demonstrates the superiority of the proposed method.

**Conclusion:**

The results showed that the nonlinear dynamical analysis based on Rcurrence Quantification Analysis (RQA) can be employed as a suitable approach for characterizing the nonlinear EEG dynamics and detecting the seizure.

## Highlights

- A method is proposed based on nonlinear dynamic analysis and mutual information.
- The method is used for distinguishing among normal, ictal, and interictal EEG.
- Recurrence quantification analysis is used to extract features of the EEG signals.
- Mutual information is employed to select the most relevant features.
- This method could distinguish normal, ictal, and interictal states with 100% accuracy.

## Plain Language Summary

Currently, there is a strong demand for developing automatic seizure detection systems. A seizure detection system must be able to identify the occurrence of seizures from the ongoing or intracranial EEG that can be achieved by classification of the brain signals. Various methods have been proposed to deal with the automatic seizure detection problem.

In this paper, a new method is proposed for automatic seizure detection. It is based on nonlinear dynamic analysis of the EEG signal and mutual information. The Recurrence Quantification Analysis (RQA) has been used for characterizing the nonlinear EEG dynamics. The study results show that a robust accurate seizure detection with short period of time (1.475 s) can be obtained using the proposed method. The method could distinguish normal, ictal, and interictal states with 100% accuracy. The results hold a promising approach to automatic seizure.

## 1. Introduction

Epilepsy is a chronic neurological disorder that can cause recurrent seizures and characterized by sudden, excessive, disordered, hypersynchronous, and localized electrical discharge of a group of neurons in the brain that can temporarily change brain functions, (i.e. transient impairments of sensation, altered state of consciousness, or loss of awareness, and focal involuntary movements or convulsions (Osorio, Zaveri, Frei, & Arthurs, 2016).

Sudden and recurrent seizures can have significant effect on the life of the epileptic patient. Obviously reliable real-time detection of seizures could significantly improve the therapeutic potentials like “closed-loop” therapies. In closed-loop therapies, electrical stimulation, drug infusion, cooling, or biofeedback may be delivered in response to seizure detection (Ramgopal et al., 2014). Patients with epilepsy are usually treated with Antiepileptic Drugs (AEDs) to control their seizures (Wlodarczyk, Palacios, George, & Finnell, 2012); accurate real-time detection of seizures is critical to reduce the side effects by on demand delivering of AEDs during the preictal phase with short-acting drugs.

A conventional technique for diagnosis and analysis of epilepsy is the long-term EEG recording for several days and then visual inspection of EEG recordings by human specialists. To reduce the burden of time-consuming inspection, a robust real-time seizure-detection system could facilitate long-term monitoring and localization of the epileptogenic zone (i.e. the brain zone that can generate seizures), which is helpful in preoperational evaluations. Accordingly, there is a strong demand for developing Automatic Seizure Detection (ASD) systems. A seizure detection system should be able to identify the occurrence of seizures from the on going EEG or intracranial EEG by classification of the brain signals. Different approaches have been proposed to deal with the automatic seizure detection. The key components of seizure detection are feature extraction from brain electrical activity and then their classification. So far, different approaches based on time-domain analysis, frequency-domain analysis, and information theory have been used for feature extraction (Thomas, Temko, Marnane, Boylan, & Lightbody, 2013; Yang, Jeannès, Bellanger, & Shu, 2013; Page et al., 2015; Peker, Sen, & Delen, 2016).

Empirical Mode Decomposition (EMD) have been used for extracting features from the Intrinsic Mode Functions (IMFs) of EEG signals for seizure detection (Pachori, 2008; Oweis & Abdulhay, 2011; Pachori & Bajaj, 2011; Bajaj & Pachori, 2012; Alam & Bhuiyan, 2013; Riaz, Hassan, Rehman, Niazi, & Dremstrup, 2016). The mean frequency measure of IMFs has been used as a feature to recognize the difference between seizure (ictal) and seizure-free (interictal) EEG signals (Pachori, 2008). Oweis and Abdulhay (2011) used the weighted frequency of IMFs as the feature set for discriminating healthy EEG from epileptic EEG signals.

The area measurement of the analytic IMFs has been also used as a feature set for discriminating healthy from the epileptic seizure (Pachori & Bajaj, 2011). Bajaj and Pachori (2012) used the amplitude and frequency modulation bandwidths of the analytic IMFs as the feature set for distinguishing seizure and non-seizure EEG signals. The higher order moments, including variance, kurtosis, and skewness, extracted from the IMFs of the EEG signals were used as the features for classification of various cases; including healthy, interictal, and ictal; healthy and seizure; nonseizure and seizure; and interictal and ictal (Alam & Bhuiyan, 2013). Recently, spectral centroid, coefficient of variation, and the spectral skew of the IMFs have been used for feature extraction to detect epileptic seizures (Riaz et al., 2016).

A series of studies have focused on Nonlinear Dynamical Analysis (NDA) of EEG signals to extract features for detection of epilepsy (Srinivasan, Eswaran, & Sriraam, 2007; Chen et al., 2011; Niknazar et al., 2013; Yaylali, Koçak, & Jayakar, 1996; Cerf, Amri, Ouasdad, & Hirsch, 1999; Adeli, Ghosh-Dastidar, & Dadmehr, 2007; Ghosh-Dastidar, Adeli, & Dadmehr, 2007; Iasemidis et al., 2003; Van Drongelen et al., 2003; Easwaramoorthy & Uthayakumar, 2011; Zhou, Liu, Yuan, & Li, 2013; Zabihi et al., 2016; Thomasson, Hoeppner, Webber, & Zbilut, 2001; Li, Ouyang, Yao, & Guan, 2004; Ouyang, Li, Dang, & Richards, 2008; Niknazar, Mousavi, Vahdat, & Sayyah 2013). These features include Approximate Entropy (ApEn) (Srinivasan et al., 2007; Chen et al., 2011; Niknazar et al., 2013), correlation dimension (Yaylali et al., 1996; Cerf et al., 1999; Adeli et al., 2007; Ghosh-Dastidar et al., 2007), Lyapunov exponent (Niknazar et al., 2013; Adeli et al., 2007; Ghosh-Dastidar et al., 2007; Iasemidis et al., 2003), Kolmogorov entropy (Van Drongelen et al., 2003), fractal dimension (Niknazar et al., 2013; Easwaramoorthy & Uthayakumar, 2011), lacunarity (Zhou et al., 2013), and features extracted from Poincaré section (Zabihi et al., 2016) as well as Recurrence Quantification Analysis (RQA) (Niknazar et al., 2013; Thomasson et al., 2001; Li et al., 2004; Ouyang et al., 2008; Niknazar et al., 2013).

In spite of numerous approaches for feature extraction, a major challenge to classify the electrical brain activity for detecting epilepsy is the selection from a large number of available EEG features. Searching important and relevant features is essential to improve the accuracy, efficiency, and generalization of a classification process. There have been a few studies on the feature selection for seizure detection (D’Alessandro et al., 2003; Temko, Nadeu, Marnane, Boylan, & Light-body, 2011; Wang & Lyu, 2015; Zhang & Parhi, 2016). Genetic algorithm (D’Alessandro et al., 2003), recursive feature elimination (Temko et al., 2011; Wang & Lyu, 2015), and Fisher’s linear discriminant analysis combined with the branch and bound algorithm (Zhang & Parhi, 2016) were employed to select EEG features for epileptic seizure detection.

In this paper, a feature selection algorithm, which is based on Mutual Information (MI) estimates (Kwak & Choi, 2002; Peng, Long, & Ding, 2005) is used for seizure detection. MI is a nonparametric measure of the dependence between random variables and is always non-negative. In terms of MI, the aim of the feature selection is to find features from a large feature set which jointly has the largest dependency on the target class. The original features were extracted from RQA of the EEG signals. The RQA of the EEG signals is used to characterize the nonlinear EEG dynamics and extract appropriate features for automatic seizure detection. We extend the previous RQA-based features and introduce different RQA measures, which are important for measuring the complexity.

**Figure 1.**
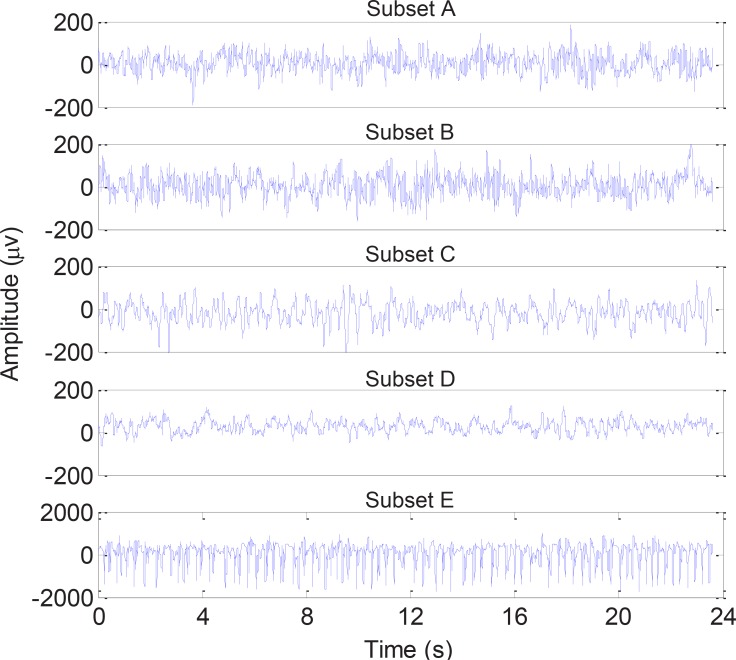
Examples of EEG signals from each of the five subsets A, B, C, D, and E

The dataset provided by Dr. R. Andrzejak was used in this study (Andrzejak, Lehnertz, Rieke, Mormann, David, & Elger, 2001). The dataset consists of 500 single-channel EEG segments, each lasts 23.6 s, and is categorized into five subsets (marked as sets A–E) while each subset contains 100 EEG segments. The subsets A and B have been recorded from five healthy candidates with their eyes open and closed, respectively, using the standard 10–20 electrode arrangement. The subsets C and D contain EEG signals recorded during interictal intervals from the epileptogenic region and the hippocampal formation of the opposite hemisphere, respectively. The subset E includes EEG segments corresponding to seizure attacks, recorded using all the electrodes. The subsets A and B have been recorded extracranially, whereas subsets C, D, and E have been recorded intracranially. The EEG signals were recorded in a digital format at the sampling rate of 173.61 Hz and were band-pass filtered between 0.53 and 60 Hz. Figure 1 demonstrates the typical EEG signals from each subset.

## 2. Methods

### 2.1. Feature extraction procedure

The original features were extracted from the RQA of the EEG signals. The first step in RQA is the reconstruction of the phase space trajectory and construction of the Recurrence Plot (RP). RP is a technique, which can visualize the recurrence of the system states of a dynamical system in the phase space.

#### 2.1.1. Phase space reconstruction

An important step in the analysis of any dynamical system is the reconstruction of its phase space. The phase space of a dynamical system is a space, which shows all states of a system, whereas each state of the system corresponds to one unique point in the phase space. Phase space is a geometrical representation of system dynamics. A frequently used method for the phase space reconstruction is Taken’s time delay method (Marwan, Romano, Thiel, & Kurths, 2007). According to Taken’s theorem, the dynamics of time series (*u_1_,u_2_,…,u_N_*) can be embedded in an m-dimensional phase space by the vector as follows:

(1) $$x_{t}=(u_{t},u_{t+\tau },\ldots ,u_{t+(m-1)\tau })$$,

where *τ* and *m* are the time delay and the embedding dimension, respectively. In order to fully capture the dynamics, an appropriate time delay and the embedding dimension should be chosen. A proper time delay is the first local minimum of the MI function (Fraser & Swinney, 1986). Cao (1997) proposed a method to define the minimum embedding dimension from a scalar time series. The method is started with a low value of the embedding dimension *m* and then increasing it until the number of false neighbors’ reduces to zero. In this paper, we used MI and Cao’ methods to approximate the time delay and embedding dimension, respectively.

#### 2.1.2. Recurrence plot

Recurrence is a substantial nature of dynamical systems (Marwan et al., 2007). Eckmann et al. (1987) introduced a method to visualize the recurrences of dynamical systems called RP (Marwan et al., 2007). To construct the RP, a symmetrical *N*×*N* array called recurrence matrix *R* is computed as follows:

(2) $$R_{i,j}(\varepsilon )=\Theta \left(\varepsilon -||{\vec{x}}_{i}-{\vec{x}}_{j}||\right)$$,

where *N* is the number of intended states *x⃗*, *Θ*(*x*) is the Heaviside function (i.e. *Θ*(*x*)=0 if *x*< and *Θ*(*x*)=1 otherwise), is the threshold distance, and ‖·‖ is a norm. Thus recurrence matrix is a matrix consisting of 1s and 0s. To calculate recurrence matrix, a suitable norm has to be selected. In this paper, we used Euclidean norm for calculating the distance between two states. RP of each dynamical system has its own topology. For example, RP related to periodic systems has uncut and long diagonal lines.

The vertical distance between these diagonal lines indicates the period of the fluctuations. The RP of chaotic system also has diagonal lines, which are shorter than periodic systems with certain vertical distances. But, vertical distances in chaotic systems are not as regular as in the periodic systems. The RP of the uncorrelated stochastic signal consists of many single black points.

#### 2.1.3. Recurrence quantification analysis

To quantify the structures in RPs, several measures of complexity have been proposed. These measures are known as Recurrence Quantification Analysis (RQA) (Marwan et al., 2007) and are based on the recurrence point density, the diagonal and vertical line structures, recurrence time, and recurrence network.

##### 2.1.3.1. Recurrence point density measure

(3) $$RR=\frac{1}{N^{2}}\sum_{i,j=1}^{N}R_{i,j}(\varepsilon )$$

The measure *RR* describes the probability that a state recurs to its *ɛ*-neighborhood in phase space.

##### 2.1.3.2. Diagonal line measures

These measures are calculated from the histogram *P*(*ɛ,l*) of diagonal lines with a length of *l*. The RP of stochastic systems has none or short diagonal lines structure and more single points, while deterministic systems are characterized by longer diagonal lines and less single isolated recurrence points.

The Determinism (*DET*) or predictability of a system can be measured by the ratio of recurrence points that form diagonal structures (of at least length *l_min_*) to all recurrence points:

(4) $$\mathrm{DET}=\frac{\sum_{l=l\min}^{N}l\cdot P(l)}{\sum_{l=1}^{N}l\cdot P(l)}$$,

where *l_min_* is the least length and *P*(*l*) is the frequency distribution of the length l of the diagonal structures in the RP. In this paper, we selected *l_min_*=2. Diagonal line length (*L*) shows that a segment of the trajectory is partly close during *l* time step to another segment of the trajectory at a different time. These diagonal lines indicate the divergence of the trajectory segments. The average time that two segments of the trajectory are close to each other can be measured by the mean diagonal line length, and can be considered as the mean prediction time (Marwan et al., 2007):

(5) $$L=\frac{\sum_{l=l\min}^{N}l\cdot P(l)}{\sum_{l=l\min}^{N}l\cdot P(l)}$$

The main diagonal is not considered for calculation. Maximal diagonal line length (*L_max_*) is the length of the longest diagonal line in the RP that is parallel to the main diagonal (Marwan et al., 2007). The main diagonal line is not considered for calculation of *L_max_*. The exponential divergence of the phase space trajectory is measured by *L_max_*. The shorter diagonal lines indicate faster trajectory divergence.

Entropy of the diagonal line lengths (*ENTR*) is calculated as follows:

(6) $$\mathrm{ENTR}=-\sum_{l=l\min}^{N}p(l)\mathrm{lnp}(l)$$,

where *p*(*l*) is the probability that a diagonal line has exactly the length *l*. Complexity in the RP can be measured by *ENTR*. The small value of *ENTR* indicates strong regularity and less complexity and the large value indicates significant fluctuations.

##### 2.1.3.3. Vertical line measures

The chaos-chaos, order-chaos, and chaos-order transitions can be found by vertical line measures (Marwan, Wessel, Meyerfeldt, Schirdewan, & Kurths, 2002). Hence, these measures are appropriate for investigating the intermittency and short and non-stationary data series. The ratio of the recurrence points forming the vertical structures to the entire set of recurrence points is defined as the Laminarity (*LAM*) as follows:

(7) $$\mathrm{LAM}=\frac{\sum_{v=v_{\min}}^{N}vP(v)}{\sum_{v=v}^{N}vP(v)}$$,

where the *P*(*v*) is the histogram of the vertical lines with length *v* and *v_min_* is minimal length.

*Trapping Time* (*TT*) is the average length of vertical structures as follows:

(8) $$\mathrm{TT}=\frac{\sum_{v=v\;\min}^{N}vP(v)}{\sum_{v=v\;\min}^{N}vP(v)}$$,

which describes the mean time that the system remains in a state.

*Maximal vertical line length* (*V_max_*) is the length of the longest vertical line in the RP:

(9) $$V_{\max}=\max\left(\left\{v_{i};i=1,\ldots ,N_{v}\right\}\right)$$,

where *N_v_* is the total number of vertical lines in RP.

##### 2.1.3.4. Recurrence time-based measures

**Figure 2.**
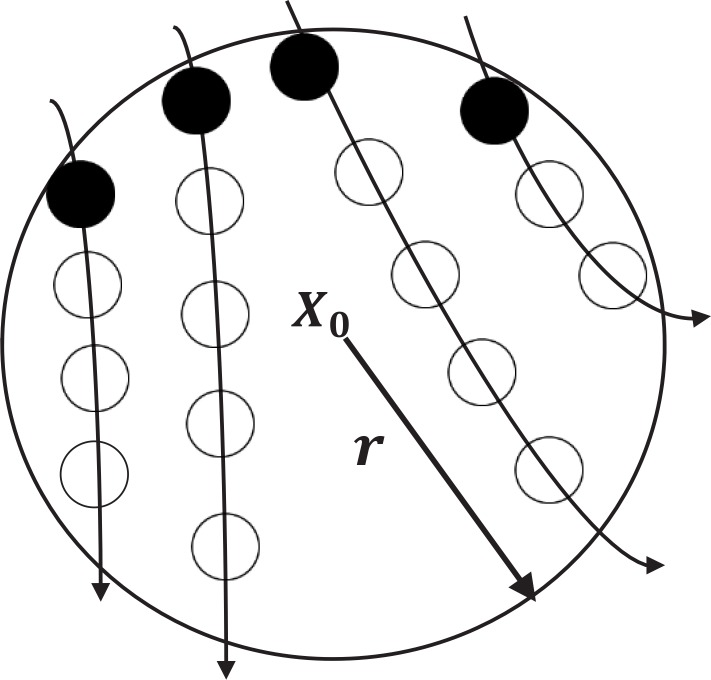
A schematic representation of the recurrence points of the second type (solid circles) and the sojourn points (open circles)

Three RQA measures based on recurrence time statistics have been proposed for detecting the transitional signals in noisy and nonstationary environments (Gao, Cao, Gu, Harris, & Principe, 2003). These measures are called the first type T_1_ and the second type T_2_ of recurrence time and Recurrence Period Density Entropy (*RPDE*). To define the second type of recurrence time, consider a scalar time series {*u*(*i*), *i*=1,2,…} and corresponding reconstructed trajectory in m-dimensional phase space as *x_t_*=(*u_t_,u_t+τ_,…, u_t+(m-1)τ_*). An arbitrary reference point (*x_0_*) on the reconstructed trajectory is selected, then a neighborhood of radius r for reference point *B_r_*(*x_0_*)={*x*:‖*x*–*x_0_* ‖≤*r*} is defined. The set of points consisting of the first trajectory point getting inside the neighborhood from outside are defined as recurrence points of the second type (Figure 2).

The trajectories that remain inside the neighborhood for a while, produce a sequence of points that are called the sojourn points (white circle in Figure 2). The set of the recurrence points of the second type as well as the sojourn points constitute the recurrence points of the first type. If the recurrence points are defined as *S*={*x_t1_,x_t2_,…,x_ti_,…*}, then the corresponding recurrence time *T* is {*T*(*i*)=*t_i+1_–t_i,i_=1,2*,…} (Figure 2).

Recurrence Period Density Entropy (*RPDE*) is a measure that can describe the complexity of a signal and determine the periodicity of a signal (Mukherjee et al., 2015). A system with periodic behavior has a *RPDE* with a value close to 0, whereas a system with chaotic behavior has RPDE a close to 1 (Nair & Kiasaleh, 2014). The *RPDE* can be computed as follows:

(10) $$\mathrm{RPDE}=-{\left(\ln T_{\max}\right)}^{-1}\sum_{t=1}^{T\max}P(t)\ln P(t)$$,

where *T_max_* and *P*(*t*) are the largest recurrence value and the recurrence period density function, respectively.

##### 2.1.3.5. Recurrence network analysis based measure

Recurrence Network Analysis (*RNA*) measure is the so-called network transitivity (*Trans*) and is based on the adjacency matrix A elements (Webber & Marwan, 2015), defined as:

(11) $$\mathrm{Trans}=\frac{\sum_{i,j,k=1}^{N}A_{i,j}A_{j,k}A_{k,i}}{\sum_{i,j,k=1}^{N}A_{i,j}A_{k,i}}$$,

where *A*=*R*–*I*. Trans reflects network complexity and distinguishes between regular and irregular dynamics.

### 2.2. Feature selection based on mutual information

The relevance between two variables can be measured by MI. A formalism for quantifying MI is Shannon’s information theory. Assume *X* is a random variable that represents continuous-valued random feature vector, and *C* is a discrete-valued random variable that represents the class labels, the MI between two variables *X* and *C* are calculated as follows:

(12) $$I(X;C)=\sum_{c\varepsilon C}\int_{x}p(c,x)\log\frac{p(c,x)}{p(c)p(x)}dx$$,

where *p*(*c*, *x*) is the joint probability density function of *x* and *c*, and *p*(*c*) are the marginal probability density functions of *x* and *c*, respectively. A large value of the MI between two random variables indicates that two variables are closely related. If two random variables are strictly independent, the MI is 0. In terms of MI, the optimal feature selection requires selecting a feature set f with m features, which jointly have the largest dependency on the target class *C* (i.e. maximal dependency). That is, we seek:

(13) $$\begin{array}{l} \max D(f,c),\;D=I(f;C)\\ I(f,C)=\sum_{c\varepsilon C}\int K\int p(f_{1}Kf_{m})\log\frac{p(f_{1}\Lambda f_{m},c)}{p(f_{1}\Lambda f_{m})p(c)}df_{1}\Lambda df_{m} \end{array}$$

However, it requires an accurate estimation of the underlying Probability Density Functions (PDFs) of the data and the integration on these PDFs. Moreover, due to the tremendous computational requirements of the method, the practical applicability of the above solution to the problems requiring a large number of features is limited. To overcome this problem, a heuristic method proposed by Peng et al. (2005), which is based on minimal-Redundancy-Maximal-Relevance (mRMR) framework. It was proven that mRMR criterion is equivalent to maximal dependency (13) if one feature is added at one time (Peng et al., 2005). This criterion is given by:

(14) $$J=\left\{I\left(f_{i};c\right)-\beta \sum_{fs\varepsilon s}I\left(f_{i};f_{s}\right)\right\}$$

According to this criterion, Term *I*(*fi;c*) indicates dependency between a new feature *F_i_* and the target class that should be maximized (*i.e. max_i_I*(*f_i_;c*) and the term *Σ_fsɛS_I*(*f_i_;f_s_*) indicates the dependency of the new feature with the already selected features. This term should be minimized (*i.e. min_i_Σ_fsɛS_I*(*f_i_;f_s_*). The parameter ß is the redundancy parameter, which regulates the relative importance of the MI between the new feature and the already selected features with respect to the MI with the output class.

### 2.3. Classification of EEG features

Each EEG segment was split into 16 blocks of 1.475 s duration. Original features were formed from each block. Thus, 1600 feature vectors were constructed from each EEG subset. Then, the MI-based feature selection process was carried out to select optimal feature vector. For classification of the selected features, a two-layer feed-forward neural network was employed to perform the classification. The scaled conjugate gradient algorithm was used to train the network using the selected feature vectors. The number of neurons in the hidden layer was 20 and the output layer was equal to the number of classes. Repeated random sub-sampling for evaluation, whereas during each repeat, 60%, 5%, and 35% of the feature vectors are randomly selected for training, validation, and testing, respectively. The evaluation procedure was repeated 20 times and the mean and standard deviation were calculated. Classifications were executed using MATLAB.

For the EEG dataset described in Section 2, five different cases of classification were considered. The cases were selected due to their clinical relevance and wide usage by the researchers (Alam & Bhuiyan, 2013; Riaz et al., 2016). In Case I, the sets A and B were grouped as healthy class, the sets C and D were grouped as interictal class, and the set E was recognized as ictal class. In Case II, the sets A, D, and E were considered as healthy, interictal, and ictal classes, respectively. In Case III, the sets A and E were classified as *healthy* and *interictal*, *ictal* classes, respectively. In Case IV, the sets A, B, C, and D were grouped as *nonseizure* class and the set E as *seizure* class. In case V, the first class consisted of the set D as *interictal* class and the second class included the set E as *ictal* class.

## 3. Results

### 3.1. RP of the EEG signals

**Figure 3.**
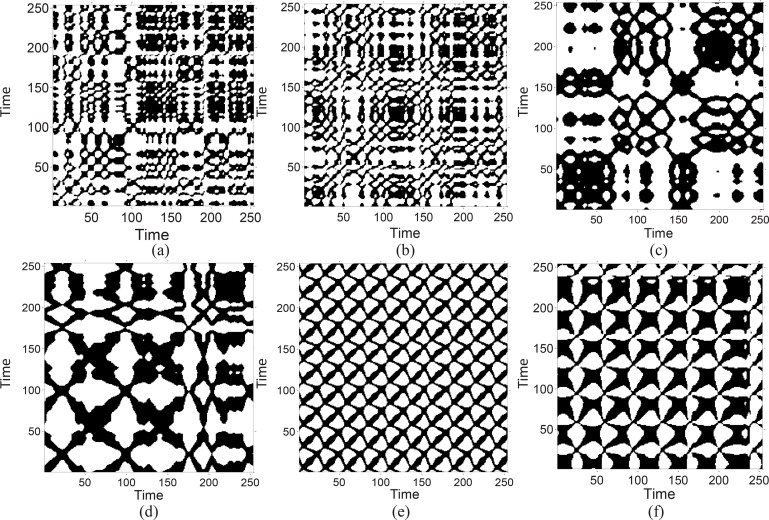
Recurrence plot of a block of subsets A (a), B (b), C (c), D (d), and E (e–f)

Figure 3 shows examples of RP of the EEG recordings corresponding to healthy (A and B), interictal (C and D), and ictal (E) conditions. It is observed that there are vertical and horizontal line structures in the RP of the healthy subject (Figure 3a and 3b). The rectangles formed by the vertical and horizontal lines indicate that the system trapped in a state and does not change or changes very slowly for some time. The vertical structures in the RP of EEG indicate intermittency and laminar. Interesting observation is the white band structures during seizure-free (Figure 3c and 3d). White area or bands corresponds to sudden changes in the dynamic as well as extreme events (Webber & Marwan, 2015). During a seizure, diagonal lines and checkerboard structures are observed in RP (Figure 3e and 3f). These structures indicate the system with periodic or quasi-periodic behavior (Webber & Marwan, 2015). The results demonstrate that the RP can visualize the dynamic changes of the EEG signals during different brain states.

### 3.2. Mutual information-based feature selection

**Figure 4.**
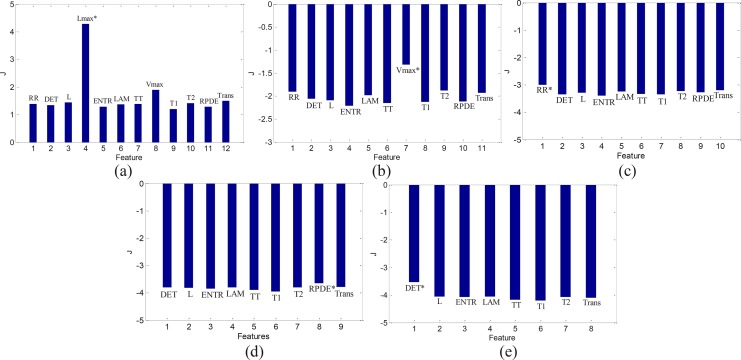
Feature selection process using mRMR algorithm for the Case I; Selection of the first (a), second (b), third (c), fourth (d), and fifth (e) feature

Figure 4 shows the results of feature selection using mRMR for the Case I. It is observed that *L_max_* is the first relevant feature that is selected (Figure 4a). According to mRMR criterion, the feature that has maximum MI with the class labels is selected as the first relevant feature. As already mentioned, *L_max_* is a RQA measure based on the diagonal lines structures and indicates repeating recurrences within a state. The diagonal lines are long for periodic signals and short for chaotic signals (Webber & Marwan, 2015). The second, third, fourth, and fifth selected features are *V_max_*, *RR*, *RPDE*, and *DET*, respectively (Figure 4b and 4e).

Table 1 summarizes the results of feature selection using mRMR for different cases of classification. It is observed that in all cases, *L_max_* is the first feature that is selected. Moreover, *V_max_* is also selected in all cases.

**Table 1.** Selected features using mRMR algorithm for different cases of classification

**Case**	**Case I**	**Case II**	**Case III**	**Case IV**	**Case V**
First selected feature	L_max_	L_max_	L_max_	L_max_	L_max_
Second selected feature	V_max_	V_max_	DET	V_max_	V_max_
Third selected feature	RR	LAM	V_max_	RR	LAM
Fourth selected feature	PRDE	T1	T1	PRDE	Trans
Fifth selected feature	DET	T2	TT	DET	T1

### 3.3. Classification

**Figure 5.**
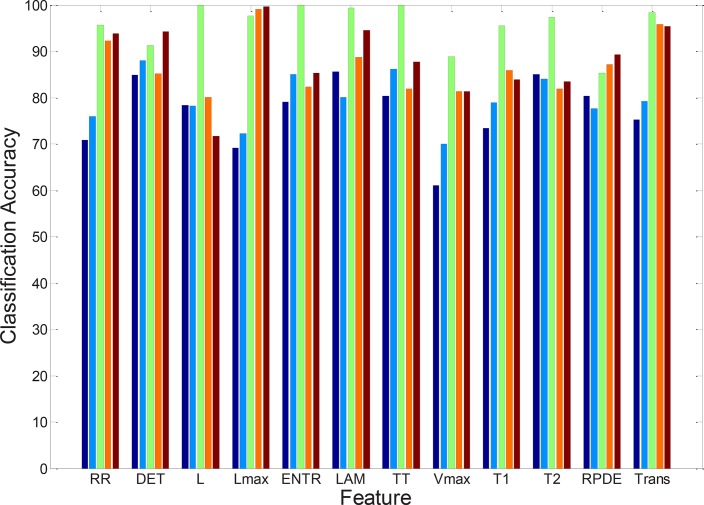
Classification accuracy of different features for different cases of classification Case I: Dark blue; Case II: Blue; Case III: Green; Case IV: Red; Case V: Brown

The classification accuracy for different RQA-based features is shown in Figure 5. It is observed that *L_max_* and Trans features provide a high classification accuracy for the Cases III, IV, and V with respect to the cases I and II. This is because the EEG data grouped into two classes in the Cases III, IV, and V while the classification Cases of I and II have three classes. Moreover, diagonal structures within the RP reflect the system with periodic and quasi-periodic behavior and the *Trans* feature can distinguish regular from irregular dynamics. The *L* could discriminate accurately Case III which contains only *healthy* and *ictal* classes.

The average overall detection accuracy, using selected features by mRMR algorithm and the feature vectors used in (Niknazar et al., 2013) is presented in Table 2. The results show that the average detection accuracy is 100% using only five selected features for all cases. In Niknazar et al. (2013), RQA was applied on the EEG recordings provided by Dr. R. Andrzejak as in the current study. The RQA-based features (i.e. *DET*, *L*, *L_max_*, *ENTR*, *LAM*, *TT*) of the original signal and subbands (i.e. delta, theta, alpha, beta, and gamma) were used for classification. The overall accuracy was 89.50% and 98.67% using the RQA-based features of the original signal (i.e. 6 features) and a combination of the original signal and subbands (i.e. 36 features), respectively.

**Table 2.** The mean of classification accuracy (± SD) for different number of selected features

**Case**	**Signal**	**I**	**II**	**III**	**IV**	**V**

**RQA-Based Features**						
Three selected features (mRMR)	Original	87.91±4.39	98.61±4.79	99.85±0.64	99.6±0.43	98.9±0.34
Four selected features (mRMR)	Original	97.59±1.52	99.28±1.52	100	100	100
Five selected features (mRMR)	Original	100	100	100	100	100
*DET, L, L_max_, ENTR, LAM, TT* (Features used in Niknazar et al., 2013)	Original	89.5±1.72	-	-	-	-
	Delta band	67.46±2.68	-	-	-	-
	Theta band	77.53±2.43	-	-	-	-
	Alpha band	63.73±3.35	-	-	-	-
	Beta band	82.73±2.26	-	-	-	-
	Gamma band	86.6±7.5	-	-	-	-
	Original+subbands	98.67±0.52	-	-	-	-

The classification accuracies obtained in this study and in the previous studies are summarized in Table 3. Only the previous studies that used the data set provided by Dr R. Andrzejak were considered for comparison to provide a fair comparison.

**Table 3.** Comparison of the results obtained by the proposed method and other methods

**Case**	**Percent**					**Number of Feature**	**Block Duration**	**Method**
	
**Authors**	**Case 1**	**Case II**	**Case III**	**Case IV**	**Case V**			
Peker et al. (2016)	98.28	99.30	100	99.33	-	5	23.6	DTCWT-CVANN
Shafiul Alam et al. (2013)	80	100	100	100	100	3	1.475	EMD-higher order moments-neural network
Riaz et al. (2016)	94	91	99	98	96	6	23.6	EMD-based temporal and spectral features
Srinivasan et al. (2007)	-	-	100	-	-	1	2.95	ApEn- neural network
Ghosh-Dastidar et al. (2007)	-	96.7	-	-	-	9	23.6	Mixed-band wavelet-chaos, Levenberg-Marquardt backpropagation NN
Niknazar et al. (2013)	98.67	-	-	-	-	36	23.6	RQA on EEG signal and its wavelet-based sub-bands-ECOC
Kumar et al. (2014)	-	-	100	97.38	95.85	6	23.6	DWT-Fuzzy ApEn-SVM
Guo et al. (2011)	-	93.5	99.2	-	-	3	23.6	Genetic algorithm-KNN
Orhan et al. (2011)	95.6				-	56	23.6	DWT-K-means clustering-probability distribution-MLPNN
		96.67				56		
			100			4		
				99.6		18		
Iscan et al. (2011)	-	-	100	-	-	10	1.475	Combined time and frequency features
Wang et al. (2011)	-	-	99.44	-	-	4	1.475	Wavelet packet entropy-hierarchical EEG classification
Naghsh-Nilchi et al. (2010)	-	97.49	-	-	-	27	23.6	Eigen-system spectral estimation-MLPNN
Subasi et al. (2010)	-	-	100	-	-	24	2.95	DWT-PCA, ICA, LDA and SVM
Guo et al. (2010)	-	-	99.6	97.77	-	5	23.6	DWT-line length feature-MLPNN
Übeyli (2009)	-	96.33	-	-	-	9	1.475	DWT-Lyapunov exponents, Eigenvector-MLP
Tzallas et al. (2009)	-	100	100	-	-	3	23.6	Time-Frequency analysis-neural network
Ghosh-Dastidar et al. (2008)	-	96.6	-	-	-	9	23.6	Wavelet-chaos, PCA-NN
Subasi (2007)	-	-	94.5	-	-	16	2.95	DWT-mixture of expert model
Güler et al. (2005)	-	96.79	-	-	-	4	1.475	Lyapunov exponent-Recurrent neural network
The current study	100	100	100	100	100	5	1.475	RQA, mutual information, neural network

## 4. Discussion

There is significant interest in developing accurate automatic seizure detection. The classification of EEG into healthy, ictal, and interictal EEGs is the main goal of seizure detection. Two major components of a classification process are the feature extraction and feature selection. Different linear approaches have been proposed for time series analysis of EEG signal and extraction features. However, the linear approaches ignore the underlying nonlinear EEG dynamics. The complex nonlinear EEG dynamics show different transitions between regular, laminar, and chaotic behaviors.

The knowledge of these transitions is necessary for characterizing the underlying dynamics. A very useful nonlinear approach for measuring the complexity of a nonlinear dynamical system is RQA. Up to now, different RQA measures, including *RR*, *DET*, *L*, *L_max_*, *ENTR*, *LAM*, *TT*, and *Trend* have been used as the features of the EEG signal for seizure detection. In the current study, different RQA measures, including *V_max_*, *T_1_*, *T_2_*, *RPDE Trans* and have been introduced as the features for the EEG classification. These measures are very important for detecting the dynamic transitions and measuring the complexity.

Moreover, a systematic approach based on MI has been proposed to select the most relevant features. The first selected feature in all cases was *L_max_*. Deterministic processes have longer diagonals and less single, isolated recurrence points, whereas chaotic signals cause the short diagonal lines. The diagonal lines for periodic signals are long and for stochastic signals are absent (Webber & Marwan, 2015).

During the interictal state, the EEG signals have lower amplitude and are less rhythmic and more irregular in morphology. During ictal state, an abrupt change in the amplitude, frequency, and morphology of the EEG signals occurs, and rhythmicity increases and a synchronization of activity happens across widespread areas of the cerebral cortex. Therefore, diagonal lines can provide a suitable measure for prediction of rhythmic and periodic EEG patterns. As it can be seen in Figure 3e and 3f, the RP of the brain signals during ictal state has the checkerboard structures indicating periodic behavior whereas such structures have not been observed during healthy and interictal states.

The second selected feature is *V_max_*, which indicates the vertical line structure in the RP. RP of the healthy signal (Figure 3a and 3b) contains vertical and horizontal lines that form rectangles. This structure indicates that some states do not change or change slowly for some time (laminar states) or the process is halted at a singularity in which the dynamic is stuck in paused states.

Another selected feature is *DET*, which is a measure of determinism. In the seizure state, excessive synchronization of large neuronal populations occurs, leading to a hypersynchronous state which implies an increasing determinism of EEG data. Therefore, *DET* can be a suitable measure for seizure detection. *Trans* and *RPDE* are other complexity measures which were selected as the features.

The results of this study show that a robust accurate seizure detection with a short period of time (1.475 s) can be obtained using the proposed method. The method could distinguish *healthy*, *ictal*, and *interictal* states with 100% accuracy.

## Ethical Considerations

### Compliance with ethical guidelines

There is no ethical principle to be considered doing this research.
